# GestationaL Obesity Weight management: Implementation of National Guidelines (GLOWING): a pilot cluster randomised controlled trial of a guideline implementation intervention for the management of maternal obesity by midwives

**DOI:** 10.1186/s40814-018-0241-4

**Published:** 2018-02-09

**Authors:** Nicola Heslehurst, Judith Rankin, Catherine McParlin, Falko F. Sniehotta, Denise Howel, Stephen Rice, Elaine McColl

**Affiliations:** 1 Institute of Health and Society, Newcastle University, Baddiley-Clark Building, Richardson Road, Newcastle upon Tyne, NE2 4AX UK; 2 Newcastle upon Tyne Hospitals NHS Foundation Trust, Newcastle upon Tyne, UK

**Keywords:** Cluster RCT, Implementation, Guidelines, Midwives, Behaviour change, Social cognitive theory, Obesity, Weight management, Pregnancy

## Abstract

**Background:**

Weight management in pregnancy guidelines exist, although dissemination alone is an ineffective means of implementation. Midwives identify the need for support to overcome complex barriers to practice. An evaluation of an intervention to support midwives’ guideline implementation would require a large-scale cluster randomised controlled trial. A pilot study is necessary to explore the feasibility of delivery and evaluation prior to a definitive trial. The GestationaL Obesity Weight management: Implementation of National Guidelines (GLOWING) trial aims to test whether it is feasible and acceptable to deliver a behaviour change intervention to support midwives’ implementation of weight management guidelines.

**Methods:**

GLOWING is a multi-centre parallel group pilot cluster randomised controlled trial comparing the delivery of a behaviour change intervention for midwives versus usual practice. Four NHS Trusts (clusters) will be randomised to intervention and control arms, stratified by size of maternity services. The intervention uses social cognitive theory and consists of face-to-face midwifery training plus information resources for routine practice. The main outcomes are whether the intervention and trial procedures are feasible and acceptable to participants and the feasibility of recruitment and data collection for a definitive trial. Target recruitment involves all eligible midwives in the intervention arm recruited to receive the intervention, 30 midwives and pregnant women per arm for baseline and outcome questionnaire data collection and 20 midwives and women to provide qualitative data. All quantitative and qualitative analyses will be descriptive with the purpose of informing the development of the definitive trial.

**Discussion:**

This pilot study has been developed to support community midwives’ implementation of guidelines. Community midwives have been selected as they usually carry out the booking appointment which includes measuring and discussing maternal body mass index. A cluster design is the gold standard in implementation research as there would be a high risk of contamination if randomisation was at individual midwife level: community midwives usually work in locality-based teams, interact on a daily basis, and share care of pregnant women. The results of the pilot trial will be used to further develop and refine GLOWING prior to a definitive trial to evaluate effectiveness and cost-effectiveness.

**Trial registration:**

ISRCTN46869894; retrospectively registered 25th May 2016.

**Electronic supplementary material:**

The online version of this article (10.1186/s40814-018-0241-4) contains supplementary material, which is available to authorized users.

## Background

First trimester maternal obesity (body mass index (BMI) ≥ 30 kg/m^2^) in England doubled from 7.6% in 1989 (approximately 45,000 women) to 15.6% in 2007 (approximately 92,500 women) [1], and data from 2015 suggests 1 in 5 women in the UK enter pregnancy with an obese BMI [2]. Maternal obesity is significantly associated with inequalities including socio-economic deprivation, ethnic minority groups, and unemployment [1, 3]. Maternal obesity has short- and long-term implications for women and babies, including maternal and neonatal mortality, gestational diabetes, thromboembolism, infection, haemorrhage, reduced breast feeding, congenital anomalies, and obesity development in offspring [4–9]. However, pregnancy is also an opportunity for intervention to address womens personal behaviour change for obesity for a number of reasons. Pregnancy is a period of metabolic plasticity; there is also a shift in attitude and spontaneous change in behaviour, making women more receptive to nutrition advice, and interventions have the potential to prevent childhood obesity among future generations [10, 11]. The National Institute for Health and Care Excellence (NICE) identifies pregnancy as being a public health opportunity for health professionals to advise and support women due to women’s increased motivation and as pregnancy is a vulnerable life stage for increased risk of excessive weight gain, weight retention and long-term obesity development [12, 13].

Maternal obesity and weight management recommendations are included in the UK and international guidelines [14, 15]. NICE evidence-based guidelines for weight management before, during and after pregnancy include recommendations for health professional advice and support including discussing obesity risks and weight-related behaviour, incorporating practical and tailored advice and being sensitive to women’s weight concerns [16]. These public health guidelines specific to weight management in pregnancy are now in the public domain and therefore available to health professionals. However, passive dissemination of guidelines alone is an ineffective means of implementing them into clinical practice and is therefore likely to reduce the chance of positive health outcomes for patients compared with more active strategies [17, 18].

Midwives have an increasing public health role and are expected to implement national guidelines into routine antenatal care. However, they also report a lack of confidence in their weight management expertise and face difficulties in discussing obesity due to its sensitive nature [19], resulting in inconsistent and ad hoc advice and variation in the level of support provided to pregnant women [1, 11, 20, 21]. Patients (including non-pregnant populations) and pregnant women with obesity have described health professionals’ (including midwives) communication as ambivalent, insulting, judgemental, insensitive and patronising [22–25]. Negative experiences have led to women avoiding or delaying accessing healthcare [11, 20] and avoiding confrontation about humiliating treatment due to fear of jeopardising maternity care [26]. Pregnant women also report that they receive inadequate information about nutrition and physical activity from health professionals and are often left confused by conflicting information [27, 28]. A systematic review has been carried out to identify published or ongoing interventions which aim to support health professionals with their maternal obesity and weight management practice. No papers meeting the eligibility criteria were identified, highlighting the importance of further research [29].

A recent report by the World Health Organisation European Office identified that health professional capacity building is required to improve maternal nutrition and offspring health [30]. They reported that health professionals needed to be trained pre- and in-service, using the latest evidence-based guidelines on nutrition, diet and physical activity, including how to develop and put into practice skills such as counselling and communication approaches to reduce stigma [30]. In the UK, guidelines published by NICE and the Royal College of Obstetricians and Gynaecologists have identified professional development as a priority area in relation to maternal obesity, recommending that health professionals should have knowledge and skills to advise on weight management and behaviour change, sensitive communication techniques and knowledge of local services [16, 31]. Midwives have also expressed the need for training and skills development to support them to overcome barriers to practice, comparing obesity with other complex sensitive topics for which structured training is available (e.g. domestic violence) [11, 19, 20]. While recognising the remit of their public health role, midwives identify a key barrier to addressing obesity and weight management in pregnancy to be a lack of knowledge, skills and confidence to do this effectively, leading to variation in the advice and support offered between midwives and maternity units [1, 21].

A future evaluation of an intervention to support health professional capacity for guideline implementation would answer the following research questions: (1) does a theory-based intervention facilitate the implementation of weight management guidelines into midwifery practice and (2) does midwifery implementation of weight management guidelines mediate obese pregnant and postnatal women’s weight and weight-related behaviours? The primary and secondary outcomes would be measures (and determinants) of midwifery practice and measures of women’s weight status and weight-related behaviours. Such an evaluation would require a large-scale cluster randomised controlled trial (RCT) where the quality of the trial can be affected by poor recruitment and retention rates; limited acceptability of, or compliance with, the intervention; and process issues in the delivery both of the intervention and of trial procedures [32]. The Medical Research Council stresses the importance of conducting pilot studies prior to large-scale trials when the intervention has multiple components [32] to reduce uncertainty and optimise the chances of a successful summative evaluation. Pilot studies can explore such potential issues to inform the design and conduct of a definitive study [33, 34]. Therefore, a necessary prerequisite, and the focus of this study, is piloting to ensure methodological rigour and scientific validity and inform the development of a protocol for a definitive trial [33]. A rehearsal (external) pilot trial of the intervention will be carried out, with integrated process evaluation. A key priority for assessment of complex interventions includes identifying how it works in everyday practice [32], which will be achieved through the process evaluation element of the rehearsal pilot trial.

## Methods

The GLOWING pilot trial is registered with the International Standard Randomised Controlled Trials Number (ISRCTN) 46869894 (Additional file 1). This protocol describes the GLOWING pilot cluster RCT using the Standard Protocol Items: Recommendations for Interventional Trials (SPIRIT) checklist (Additional file 2).

### Aim

The *G*estationa*L O*besity *W*eight management: *I*mplementation of *N*ational *G*uidelines (GLOWING) pilot study aims to test whether it is feasible and acceptable to deliver a theory-based behaviour change intervention to support midwives in overcoming barriers to practice and to facilitate the implementation of weight management guidelines. The specific pilot study objectives are to:

1. Pilot the intervention delivery, data collection and analysis methods to assess feasibility and acceptability thereof
2. Explore the intervention’s active ingredients (in success or failure) through process evaluation
3. Collect baseline and outcome data required to inform sample size estimations and scope data collection procedures for economic evaluation within the definitive trial

### Aim Design and setting

**Fig. 1:**
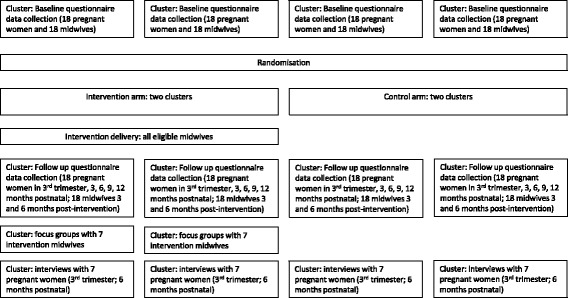
Overview of cluster trial design

GLOWING is a multi-centre parallel group cluster RCT comparing the delivery of a theory-based behaviour change intervention for community midwives versus usual practice. Usual practice is the most relevant comparator and equivalent to ‘standard care’ in patient-facing studies. The clusters are four NHS Trusts which provide maternity care in the North East of England, UK (see Fig. 1).

### Intervention

The intervention development followed a four-step approach for developing theory-informed interventions designed to facilitate the development of interventions to change clinical practice [35]. The four steps comprised: (1) identification of what needs to be changed and by whom; (2) identification of barriers and enablers addressed by the intervention using a theoretical framework; (3) identification of intervention components and modes of delivery that would help overcome barriers and enhance enablers; and (4) determining how the behaviour change can be measured and understood. Key barriers to guideline implementation best fit with the social cognitive theory (SCT), and this is the behaviour change theory used to develop the GLOWING intervention. SCT is based on the principles that the person, environment, and behaviour all interact and influence one another and that behaviours are directly related to an individual’s behavioural goals [36]. The theoretical construct at the core of the personal factors in the SCT is midwives’ self-efficacy; additional constructs include outcome expectancies and goals. The GLOWING intervention content, materials and outcome measures were developed using a systematic approach and best-available evidence. Sources included methods of behaviour change aligned to SCT [37], a systematic review of barriers and facilitators to practice which had synthesised evidence on health professionals perspective of appropriate ‘training’ modes of delivery and behaviour change techniques [19], evidence from nine related EPOC Cochrane reviews on intervention methods to change health professional behaviours [38–46] and existing behaviour change technique taxonomies [47, 48].

The details of the intervention are reported using the template for intervention description and replication (TIDieR) checklist and guide [49] (see Additional file 3). The GLOWING intervention consists of a one-off full day of intensive face-to-face training for small groups of eligible midwives (*n* = 6 per session), plus the provision of training resources for their continued professional development and information resources for midwives to share with pregnant women during routine antenatal contacts. The face-to-face intervention sessions will be delivered by a research midwife in either the local NHS Hospital Trust education facility or in a community setting with appropriate facilities for group-based learning (such as computer, projector, seating and tables). The intervention is divided into five sessions: introduction, weight communication, weight management, consolidation of the day, and summary and evaluation. The sessions include a combination of didactic and interactive activities (lectures, watching a video of a midwife booking appointment, individual and group reflection, group discussions, scripted role plays, action planning and coping planning) and will be delivered as graded tasks throughout the intervention delivery (1-day training). Graded tasks start with small tasks to gain confidence, with each subsequent task building on the previous one. The midwives attending the GLOWING sessions will be provided with a training pack including all of the intervention materials (e.g. lecture slides, reflection activities), plus additional resources to support their practice such as the NICE guidelines and relevant British Dietetic Association ‘Food Factsheets’*.* The intervention arm clusters will also be provided with a 1 year supply of written resource packs for midwives to share with pregnant and postnatal women with a booking BMI ≥ 30.0 kg/m^2^. These packs include a combination of existing nationally available patient-facing resources on maternal obesity and weight management (e.g. booklets produced by Tommy’s the Baby Charity, Start4Life, First Steps Nutrition Trust) and resources developed specifically for GLOWING, including leaflets detailing reliable national evidence-based information sources (e.g. NHS Choices, First Steps Nutrition Trust, British Dietetic Association, the EatWell Guide) and details of local support available to women in the pilot site areas (e.g. local exercise classes for pregnant or postnatal women, local websites with further information on support services). The intervention has been developed to be delivered consistently for each GLOWING session, and the facilitator will have a pack and script to use during the intervention delivery with the aim of standardising the content and delivery of each intervention session with midwives. However, the interactive nature of the intervention will require the facilitator to be responsive to the midwives’ discussions and questions on the day which makes it difficult to estimate some of the timings required for each training component. Therefore, the first GLOWING session will be used to pilot the timing of the intervention delivery, and the intervention timings will be adapted if necessary for the remaining sessions.

### Participants, inclusion and exclusion criteria

The intervention and control midwifery participants are community midwives within the trusts (clusters) as the guidelines are most relevant to this population of health professionals who have the predominant public health role in pregnancy. Hospital-based midwives with a specific obesity or weight management role will also be included. Hospital-based midwives without a specific maternal obesity or weight management role and non-midwifery health professionals will be excluded.

The intervention does not target pregnant women directly. However, if the intervention is effective and midwives implement guidelines into routine practice, then this would result in evidence-based provision of information, advice and support to help pregnant women achieve nutritionally healthy diet and physical activity behaviours. Pregnant and postnatal women with pre-pregnancy obesity will be included as participants only for the purposes of piloting outcome data collection and to provide data for the process evaluation. Pregnant women will be included if they have a booking BMI ≥ 30 kg/m^2^ (proxy measure for pre-pregnancy BMI), are ≥ 18 years of age (teenage pregnancies require specific nutritional support), have a singleton pregnancy (multiple pregnancies require specific nutritional support) and if they have had their 12-week ultrasound scan and their pregnancy has progressed beyond the high-risk period for miscarriage. Pregnant women will be excluded if they have a medical condition other than obesity which requires them to receive specialist weight management advice for that condition (e.g. women with pre-gestational diabetes who are attending a specialist antenatal diabetes clinic, pregnant women who have had bariatric surgery and require specialist nutritional support) and those with substance misuse or known cause for concern (e.g. domestic violence).

Inability to speak or read English is an exclusion criterion for both midwives and pregnant women as data will be collected by questionnaires which lack validation in non-English language.

### Sample size and randomisation

Sample sizes for pilot studies are typically not informed by formal power calculations, but rather by pragmatism and resource constraints; it has been proposed that outcome data from approximately 30 participants per arm is adequate [33]. Four clusters will be recruited with two per trial arm. The aim is to have outcome data from 30 midwives and 30 pregnant women per trial arm, and to allow for dropout, we will recruit 18 midwives and pregnant women per trust.

Computer randomisation of trusts to intervention or control arms will be performed by a statistician (DH) using anonymised unique IDs to prevent allocation bias. Randomisation will be stratified by size of the maternity service within the NHS Trust (large and small trusts will be categorised based on the number of bookings/year). Computer randomisation of unique ID codes will also be carried out within each cluster to select midwives and pregnant women to provide baseline, outcome and process evaluation data. We aim to recruit seven midwives and women in each trust for qualitative interviews and focus groups, with the aim of obtaining data from 10 midwives and women per trial arm. The random sampling and recruitment procedures will continue until the target sample sizes are reached. Random sampling has been chosen for the qualitative research as an attempt to avoid engaging with only those midwives with a specific interest in the topic, as the process evaluation requires the exploration of positive and negative perspectives. Baseline data collection (midwives and pregnant women) will be carried out before cluster randomisation (i.e. allocation concealment prior to consent and baseline data collection). Written informed consent will be obtained from midwives and pregnant women prior to their involvement in any study-specific procedures.

Due to the nature of the intervention, it is not possible for the intervention delivery team or midwives to be blinded. However, some degree of blinding will be utilised when possible, including the use of unique ID codes to blind the statistician to the clusters and midwife and pregnant women participant details during randomisation; the pregnant women will not be informed by the researchers whether their midwives are in the intervention or control arm of the study, and midwives will be asked not to disclose this information to women during their consultations. Midwives will not be aware which arm of the trial their NHS Trust has been allocated to when consenting and providing baseline data.

### Recruitment

The four NHS Trusts (clusters) which have agreed to participate in the GLOWING pilot trial agreed not to deliver any structured training on maternal obesity or weight management during the trial period, over and above their usual mandatory training for routine clinical care (e.g. it was permissible to include usual mandatory training on the local clinical care requirements relating to obesity such as BMI criteria for gestational diabetes screening). Eligible midwives within the clusters will be identified by the research midwife teams within the NHS Trust clusters who will screen staff lists and assign a unique ID to all potential midwife participants. There will be three stages of recruitment and consent for midwives: (1) we aim to recruit and consent all eligible midwives in the intervention clusters to participate in the intervention sessions (intervention arm only); (2) a random subset of midwives who participate in the intervention sessions will be selected and approached to participate in focus groups as part of the process evaluation (intervention arm only); and (3) a random sample of all eligible midwives in each cluster will be invited to provide baseline and follow-up questionnaire data (control and intervention arms).

Screening for eligible pregnant women will be carried out by the research midwife teams in each cluster using the antenatal booking data recorded in the electronic patient records, and all such potential pregnant women will be assigned a unique ID. A random sample of pregnant women in each cluster will be selected to be approached at their 20-week scan appointment for recruitment and consent to provide questionnaire data at baseline (before the intervention delivery). A random sample of different pregnant women will be selected to be approached for recruitment and consent to provide questionnaire data following the intervention delivery and at subsequent data collection time points up to 1 year postnatal. A different population of pregnant women is required as the women who provide data pre-intervention will have delivered by the time we are ready to collect post-intervention data. The purpose of baseline data collection is to explore clustering of behaviours at baseline rather than to explore change in women’s behaviours pre- and post-intervention. A random subset of post-intervention pregnant women from each cluster will be asked to provide qualitative process evaluation data. Due to the potential burden of data collection without any direct benefit of participation in the study, women will receive a £10 high street gift voucher for every data collection episode at baseline and follow-up.

Recruitment and consent of midwives and pregnant women will be carried out by the researchers identified in the delegation log at each cluster, primarily the research midwife teams or the chief investigator (NH), who have Good Clinical Practice certificates and have received training in informed consent. All potential midwives and pregnant women approached for recruitment will receive an information sheet and have the opportunity to discuss the research before deciding whether to consent to participate (see Additional file 4 for information sheets and consent forms).

### Participant withdrawal

Should any midwives or pregnant women decide to withdraw from the study, efforts will be made to report the reason for withdrawal. Midwife withdrawal could occur at any phase of involvement including participating in the intervention sessions, questionnaire completion or focus group participation. If any midwives withdraw part-way through the intervention training sessions (e.g. due to family emergency) or if they do not turn up (e.g. due to illness), then they will be offered the opportunity to attend another session to complete their training. If a midwife requests to withdraw from questionnaire or focus group data collection then the following actions will be taken: (1) for requests to completely withdraw from the study, no further data will be collected, but we will retain data already provided and used in the analysis; (2) for requests to withdraw due to change in circumstances (e.g. retirement rather than not wanting to participate further), we will retain data already provided and attempt to collect exit data (final outcome data) at the point of withdrawal and any data provided will be used in the analysis; and (3) for non-explicit withdrawal (e.g. non-return of questionnaire), up to three repeat attempts will be made to collect this data. The withdrawal of pregnant or postnatal women could be due to the researcher’s or women’s decision. Adverse pregnancy events (unrelated to the intervention) that would result in automatic withdrawal would be the death of the woman or baby (including miscarriage, late fetal loss, stillbirth, neonatal death and infant death up to 1 year postnatal follow-up) to avoid causing unneccessary distress to women or their families. For explicit or non-explicit withdrawal from data collection, the same actions will be taken as for midwives.

### Measures

The main outcomes of the pilot study relate to whether the intervention and trial procedures are feasible and acceptable to midwives and pregnant women, to assess the feasibility of collecting the outcome measures required for a definitive trial and to prioritise which outcomes should be primary or secondary outcomes for a definitive trial. An overview of the planned participant timeline is presented in the SPIRIT flow diagram (Table 1).

**Table 1:** Schedule of enrolment, interventions and assessments (participant timeline)

	Baseline (pre-intervention and pre- cluster allocation)	Cluster randomisation	Intervention delivery	1 month post- intervention	3 months following intervention delivery (to all midwives)	6 months following intervention delivery (to all midwives)	3 months postnatal	6 months postnatal	9 months postnatal	12 months postnatal
Maternity electronic patient records (all clusters)	Eligible pregnant women’s characteristics									
Random sample of 18 women attending 20-week scan/cluster (all clusters)	Eligibility screening, consent, questionnaire data. Clinical audit (to be carried out after delivery)									
Random sample of 18 midwives/cluster (all clusters)	Eligibility screening, consent, baseline questionnaire data				Follow-up questionnaire data	Follow-up questionnaire data				
All community midwives (intervention arm only, 2 clusters)			Consent, intervention delivery, completion of evaluation forms							
Random sample of 7 midwives/cluster (intervention arm only, 2 clusters)				Consent, focus groups						
Random Sample of 18 women/cluster (all clusters)						Eligibility screening, consent, 3rd trimester questionnaire data, weight measurement	Questionnaire data, weight measurement, clinical audit	Questionnaire data, weight measurement	Questionnaire data, weight measurement	Questionnaire data, weight measurement
Random sample of 7 recruited women/cluster (all clusters)						Consent, semi-structured interviews (3rd trimester)		Semi-structured interviews		

The primary outcome measures for the pilot trial are:

1. Recruitment rate of midwives attending the intervention training day in the intervention arm, calculated as a percentage of all eligible midwives invited to attend the GLOWING intervention (training) session
2. Feasibility of intervention delivery, calculated as the number of intervention sessions delivered with the planned number of midwives (six/session) in attendance at each session
3. Intensity of intervention delivery, calculated as the number of intervention sessions required to deliver the intervention to all recruited midwives in the intervention arm at the end of intervention delivery
4. Time required for intervention delivery, calculated for both intervention sites as the number of weeks from the first contact with the site to arrange the delivery of the intervention, and the delivery of the final intervention session
5. Fidelity of intervention delivery, calculated as the frequency of the delivery of the intervention as planned measured by direct observation and video recording of the intervention sessions, and frequency of deviation from protocol

The secondary outcome measures are:

1. Process evaluation of the content resources, and delivery of the intervention, to be measured by:
  - Direct observations and video recordings of the intervention delivery for all intervention sessions
  - Evaluation forms to be completed by midwives attending the intervention, on the day of the intervention
  - Focus groups with a sample of midwives who have received the intervention, 1 month after intervention delivery
2. Process evaluation of the implementation of guidelines into routine midwifery practice following the intervention, to be measured by:
  - Midwife questionnaires at baseline (pre-intervention) and 3 and 6 months after intervention delivery, including characteristics (number of years practice, speciality, ethnic group, age and gender), self-reported routine practice specific to the NICE guideline recommendations, SCT constructs of the guideline behaviours (self-efficacy, outcome expectancies, goals/intentions), knowledge of the guideline recommendations, Beliefs About Obese People (BAOP) scale [50], vignettes of different scenarios they might encounter in routine practice (simulated practice)
  - Focus groups with a sample of midwives who have received the intervention, 1 month after intervention delivery
3. Women’s experience of midwifery care relating to their weight and implementation of midwifery advice and support into their own behaviours, among women with obesity (BMI ≥ 30 kg/m^2^) receiving care from midwives, to be measured by:
  - Questionnaires at baseline pre-intervention and at follow-up post-intervention: women’s socio-demographic information (including maternal age, gestation, BMI, ethnic group, parity, employment status, qualifications, postcode), self-report of their midwives’ practice specific to the guideline recommendations (developed to reflect the midwives’ self-reported routine practice behaviour measurements), food frequency questionnaire (validated in pregnancy) [51], pregnancy physical activity questionnaire (validated in pregnancy) [52], psychosocial measures for understanding weight-related behaviours in pregnant women questionnaire (validated in pregnancy) [53], therapeutic alliance with midwives using the Health Care Alliance Questionnaire (validated in pregnancy) [54] and quality of life (EQ5D 5L)
  - Weight measurements in the third trimester and at 3, 6, 9 and 12 months postnatal
  - Semi-structured interviews in the third trimester and at 6 months postnatal
4. Data required for sample size estimations for a future definitive trial will be measured by:
  - Calculating variance of the intervention primary and secondary outcome measures (data collection by questionnaires) for the midwives at baseline (pre-intervention) and follow-up (3 and 6 months after intervention delivery)
  - Calculating variance of the intervention primary (weight measurements) and secondary (questionnaire) outcome measures for the pregnant women at baseline (3rd trimester) and follow-up (3, 6, 9 and 12 months postnatal)
  - Rates of recruitment and attrition of midwives and pregnant women who enrol in the study at the end of the study period
5. Clinical audit: data required to inform economic evaluation for a future definitive trial will be measured by an audit of routine antenatal data collection of participating women using handheld and electronic medical records to inform the variables to be included in the development of a framework for economic modelling and variables not routinely recorded which require further data collection.
6. Maternity electronic patient records of pregnant women’s characteristics including maternal age, BMI, ethnic group, parity, employment status and socio-economic status (determined by postcode anonymised by data linkage with index of multiple deprivation data) will be retrieved from the electronic patient records for all eligible women in their third trimester during the recruitment period. Data will be anonymised and aggregated to compare with recruited women to determine any participation bias for each site.

### Follow-up

Midwives will be followed up for 6 months after delivery of the intervention. Pregnant women recruited to provide post-intervention questionnaire data will be followed up for 1 year postnatally.

### Analysis

All quantitative and qualitative data analyses will be descriptive with the purpose of informing the development of the definitive trial. Analysis will include recruitment and retention rates, feasibility and fidelity of intervention delivery (i.e. have all components of the intervention been delivered as planned), any intervention organisation and delivery requirements, data collection procedures for a definitive trial, data requirements, timescales required and procedures for data manipulation for a definitive trial (e.g. data entry or coding requirements for qualitative and quantitative data).

Thematic analysis will be used for the qualitative focus group (midwives) and interview (pregnant women) data. The analyses will be used to inform the further development and refinement of the intervention content, delivery and data collection methods prior to a definitive trial.

The data analysis of the quantitative outcome measures will be descriptive (percentages, means and standard deviations or 5-number summaries [i.e. the minimum, lower quartile, median, upper quartile and maximum] as appropriate), with the primary aim of providing estimates of key trial parameters, including rates of recruitment, retention, data completion and the variability of proposed outcome measures. These key trial parameters will be used to inform power calculations for the definitive trial and to inform decisions about which outcome measures to use in the definitive trial (e.g. are any time points for maternal weight status consistently complete or incomplete, what routine data we can yield from the clinical audit etc). Estimates required to inform power calculations for a definitive trial are:

- An estimate of the variability (standard deviation) for continuous outcome measures
- An estimate of proportions (percentages) for categorical outcome measures
- An estimate of the intracluster correlation (ICC) for these measures to inform the power calculation; though given the imprecision in this estimate due to the small sample size, other sources of estimates will also need to be explored (e.g. drawing upon ICCs reported in similar studies).

### Data management, monitoring and adverse events

This is a low-risk pilot trial, and major safety issues are not anticipated. The project advisory group (PAG) includes independent members (described in the Acknowledgements) and will be responsible for monitoring the conduct of the pilot trial. The full PAG will have one pre-intervention meeting, one meeting following intervention delivery and one meeting annually until the end of the study. Additional monitoring of study conduct and data collected will be performed by a combination of central review and site monitoring visits (NH) to ensure the site files are maintained and the study is conducted in accordance with Good Clinical Practice (GCP). The study may be subject to inspection and audit by the sponsor NHS Trust and other regulatory bodies to ensure adherence to GCP.

The intervention in this pilot study is targeted exclusively at midwives. Therefore, any intervention adverse events will not involve pregnant women, and adverse events related to the pregnant women will not be captured by the study. However, adverse events which would result in automatic withdrawal of pregnant or postnatal women (unrelated to the study) will be checked before each data collection contact. There is low risk of adverse events relating directly to the intervention (midwifery training). There is a minimal possibility that midwives may become upset during the training (e.g. being asked to reflect on their past practice experiences if there has been a particularly negative experience). In this circumstance, the following procedures will be put in place: stop the intervention delivery, ask the midwife if she would like to leave the room for a while (supported by the observer who will be present during the intervention delivery), remind the midwife of her option to withdraw from the intervention, signpost to follow-up staff support in their local NHS Trust if necessary (this would involve suggesting that they contact their allocated supervisor of midwives or line manager for further support) and any instance of adverse events will be recorded in the study file at the related site.

All primary data collection will be confidential and comply with the Data Protection Act (1998), and data will be anonymised using the midwife and pregnant women’s unique study ID number. No personal data will leave the NHS clusters without explicit consent and only when absolutely necessary (e.g. names and phone numbers required for University researchers to arrange interviews with pregnant women). All paper-based study records will be kept in a locked filing cabinet with restricted access, and electronic data on a secure network folder, password-protected with restricted access. Double data entry will be used for all questionnaires to minimise data entry errors. All data will be subject to validity checks before analysis (e.g. to identify missing data, valid data ranges etc).

## Discussion

This pilot study has been developed to test the feasibility and acceptability of delivering and evaluating an implementation intervention for maternal obesity and weight management, targeting community midwives’ routine clinical practice behaviours, using a cluster RCT design. Community midwives have been specifically selected due to the majority of the NICE recommendations being related to the booking appointment (first antenatal appointment with a health professional) [16], and it is usually the role of the community midwife to carry out this consultation. Community midwives also have a key public health role as they usually have the most contacts with women throughout the antenatal period. However, some NHS Trusts also have hospital-based midwives with a specific maternal obesity or weight management role (e.g. specialist public health midwives, midwives working within antenatal obesity clinics etc), and therefore, these selected midwives have also been included. We acknowledge that other health professionals are also likely to need support to provide evidence-based obesity and weight management support for pregnant women, such as other midwife specialities, obstetricians, health visitors, GPs, maternity and health care assistants and student health professionals. Some of these specialties report similar barriers to practice to midwives in the evidence base which has been used to inform the development of the GLOWING intervention [19], and therefore, the intervention may be transferrable to other disciplines. However, further exploration of the feasibility of delivering the GLOWING intervention to additional disciplines would be required.

A cluster design is the gold standard in implementation research due to the significant risk of contamination between control and intervention groups when randomising at individual level [55]. In this pilot trial, there is risk of contamination if either midwives or pregnant women are randomised at the individual level. Randomising pregnant women within the NHS Trusts to receive advice and support relating to obesity and weight management would not be feasible as it would require the midwives to ‘switch on’ their behaviours for those women randomised to the intervention arm and to ‘unlearn’ their behaviours for women randomised to the control arm. Randomising individual midwives to intervention and control arms would also be unfeasible. Community midwives within NHS Trusts usually work in locality-based teams out in the local community, sometimes within the same GP practices, and interact on a daily basis. The risk of contamination between those working closely together would be high using individual randomisation of midwives, especially relating to the control midwives’ access to the training materials and the women’s resources in routine practice. Community midwife teams also share care of pregnant women, and any one pregnant woman could potentially see a different community midwife from the same team at each antenatal visit and occasionally from other teams within the NHS Trust. Therefore, there could also be contamination of control midwives accessing the women’s resources during routine care.

The shared care between midwives is also the reason for aiming to recruit all eligible midwives to participate in the intervention sessions, even though the target sample size for data collection is only 30 midwives per arm. If all midwives participate in GLOWING, it should increase consistency of advice and support for women. Data will be collected from pregnant and postnatal women about their midwives’ provision of advice and support around obesity and weight management, and this would also require all midwives to participate in the intervention to guarantee that the women are reporting the behaviours of GLOWING intervention midwives.

The intervention targets maternal obesity, although we acknowledge that women with a booking BMI < 30 kg/m^2^ are also likely to benefit from weight management support from community midwives. The NICE guideline recommendations on healthy diet and physical activity behaviours and managing weight in pregnancy are relevant to women with a BMI ≥ 18.5 kg/m^2^ (including those with a recommended BMI, overweight and obese), although the guidelines specify that they are particularly relevant to women with a BMI ≥ 30 kg/m^2^ [16]. Midwives can apply the changes in practice to women with a BMI < 30 kg/m^2^, but the intervention is only providing the women’s resource packs to the highest risk population of women with obesity.

The data collection and analysis during the pilot trial will determine if it is feasible and acceptable to deliver GLOWING as planned to all eligible midwives in the intervention arm. The results of the GLOWING pilot trial will be used to further develop and refine GLOWING where necessary prior to a definitive trial to evaluate the effectiveness and cost-effectiveness of the intervention.

## Additional file

Additional file 1: A pilot study to test the delivery of midwife training sessions on obesity and weight. (DOCX 30 kb)

Additional file 2: SPIRIT 2013 Checklist: Recommended items to address in a clinical trial protocol and related documents*. (DOCX 36 kb)

Additional file 3: The TIDieR (Template for Intervention Description and Replication) Checklist* Information to include when describing an intervention and the location of the information. (DOCX 20 kb)

Additional file 4: GLOWING participant information sheets and consent forms for midwives (questionnaire data collection, participating in the intervention training days, focus groups) and pregnant women (baseline data collection, outcome data collection, interviews). (DOCX 218 kb)

## Abbreviations

BMI: Body mass index
EPOC: Effective Practice and Organisation of Care
GLOWING: GestationaL Obesity Weight management: Implementation of National Guidelines
ICC: Intracluster correlation
ID: Identifier
NHS: National Health Service
NICE: National Institute for Health and Care Excellence
RCT: Randomised controlled trial
SCT: Social cognitive theory
SPIRIT: Standard Protocol Items: Recommendations for Intervention Trials
TIDieR: Template for Intervention Description and Replication
