# Supplementary material for: GestationaL Obesity Weight management: Implementation of National Guidelines (GLOWING): a pilot cluster randomised controlled trial of a guideline implementation intervention for the management of maternal obesity by midwives
Source: Pilot Feasibility Stud. 2018 Feb 9;4:47. doi: 10.1186/s40814-018-0241-4 (PMC5807844; doi:10.1186/s40814-018-0241-4)
Supplement: Supplementary file 1 — A pilot study to test the delivery of midwife training sessions on obesity and weight. (DOCX 30 kb) [file 40814_2018_241_MOESM1_ESM.docx]

**A pilot study to test the delivery of midwife training sessions on obesity and weight**

Condition category: Nutritional, Metabolic, Endocrine

Date applied: 11/04/2016

Date assigned: 25/05/2016

Last edited: 25/05/2016

Prospective/Retrospective: Retrospectively registered

Overall trial status: Ongoing

Recruitment status: Recruiting

**Plain English Summary**

*Background and study aims*
Obesity during the first 12 weeks of pregnancy (first trimester maternal obesity) in England has doubled between 1989 and 2007. The highest amount of these women tend to live in the most deprived areas of the UK, are Black and South Asian women or those who are unemployed. Maternal obesity bits both the mother and baby at risk. The most extreme risk is the death of the mother and the baby, however other risks include the development of gestational diabetes (a type of diabetes only present during pregnancy), thromboembolism (blood clots, infection, post-partum haemorrhage (bleeding after the birth), reduced breast feeding rates, birth defects, and obesity development in child. National guidelines recommend that pregnancy is a key time for health professionals to advise and support women as there is evidence that unhealthy lifestyles and excessive weight gain can contribute towards the development of obesity among women who do not start their pregnancy with obesity. Management of maternal obesity is included in numerous guidelines, and midwives are expected to support women with their weight as part of their public health role. UK national guidelines for weight management before, during and after pregnancy include recommendations for health professionals to provide advice and support to obese women. However, research has shown that health professionals lack confidence in their own expertise to provide advice and support for obese women during pregnancy, and face difficulties in discussing obesity due to its sensitive nature. Midwives have expressed the need for training and skills development to address the difficulties faced in their clinical practice which lead to inequalities in the support they provide for obese women. This study involves a training programme using a behaviour change theory called Social Cognitive Theory (a theory stating that when people see someone performing behaviour and the consequences, they remember the information and use it to guide their own future behaviour) to support midwives to implement weight management guidelines into their routine practice. The aim of this study is to use a small group of NHS Trusts to test whether it is possible to deliver the training programme on a larger scale across the UK.

*Who can participate?*Midwives at participating hospitals, and obese pregnant women over the age of 18.

*What does the study involve?*The midwives in the NHS Trusts randomly allocated to one of two groups. Those in the first group continue with the normal practice. Those in the second group receive a one day training programme, and information resources to support their routine clinical practice. Midwives in all four NHS Trusts are asked to complete questionnaires about their routine practice before the programme is delivered, and again at 3 and 6 months after training. Midwives who have received the training are also invited to take part in a focus group to share their experiences. Pregnant women are asked to complete a questionnaire about their lifestyle and about the information they have received from their midwives before the programme is delivered. After the training, pregnant women are asked to repeat the questionnaire and have their weight measured in their third trimester (39-40 weeks), and again at 3, 6, 9, and 12 months after they have had the baby. Women are also invited to have an interview to discuss the information they have received from their midwife, and their diet and physical activity behaviours in their third trimester of pregnancy and at 6 months after giving birth.

*What are the possible benefits and risks of participating?*The potential benefits for midwives include their continued professional development relating to receiving training (the need for training on this topic has been identified as a priority for midwives), as attending the training can contribute towards midwives continued professional development portfolio. Potential benefits also relate to midwives following best evidence-based practice guidelines if the project is successful, which should also benefit the pregnant women who receive care from midwives who have received the training. There are no notable risks involved with taking part in this study.

*Where is the study run from?*Four NHS Trusts located in the North of England (UK)

*When is the study starting and how long is it expected to run for?*September 2015 to September 2017

*Who is funding the study?*National Institute for Health Research (UK)

*Who is the main contact?*Dr Nicola Heslehurst, [nicola.heslehurst@ncl.ac.uk](mailto:nicola.heslehurst@ncl.ac.uk)

**Type:** Public

**Primary contact:** Dr Nicola Heslehurst

**Contact details:** Institute of Health & Society, Newcastle University, Baddiley-Clark Building, Richardson Road, Newcastle upon Tyne, NE2 4AX, United Kingdom, +44 0191 208 3823, [nicola.heslehurst@ncl.ac.uk](mailto:nicola.heslehurst@ncl.ac.uk)

**Protocol/serial number:** 20492

**Study information**

**Scientific title:** GestationaL Obesity Weight management: Implementation of National Guidelines (GLOWING): A pilot cluster randomised controlled trial of a guideline implementation intervention for the management of maternal obesity by midwives

**Acronym:** GLOWING

**Study hypothesis:** Pilot study aim: To pilot a theory-based intervention to facilitate the implementation of weight management guidelines into midwifery practice
Pilot study objectives:
1. Pilot the intervention delivery, data collection and analysis methods to ascertain feasibility and acceptability thereof
2. Identify the intervention’s active ingredients (in success or failure) through process evaluation
3. Collect baseline and outcome data required to inform sample size estimations and scope data collection procedures for economic evaluation within the definitive trial

**Ethics approval:** Proportionate Review Sub-committee of the Yorkshire & The Humber - South Yorkshire Research Ethics Committee, 16/12/2015, ref: 15/YH/0565

**Study design:** Randomised; Interventional; Design type: Process of Care, Education or Self-Management, Gene Therapy, Psychological & Behavioural, Management of Care

**Primary study design:** Interventional

**Secondary study design:** Randomised controlled trial

**Trial setting:** Not specified

**Trial type:** Treatment

**Patient information sheet:** Not available in web format, please use the contact details below to request a patient information sheet

**Condition:** Specialty: Reproductive health and childbirth, Primary sub-specialty: Reproductive and sexual medicine; UKCRC code/ Disease:

**Intervention:** Clusters (NHS Trusts providing maternity services) will be randomly allocated to intervention or control arms, stratified by size of cluster, using computer randomisation methods. Participants are community midwives or hospital-based midwives with a specific maternal obesity or weight management role in each cluster.
The intervention uses Social Cognitive Theory, and is an intensive one day training programme for midwives on weight communication and weight management in pregnancy, and the provision of information resources to support midwives clinical practice.
Control sites will receive no intervention or resources (usual practice).
Midwives will be followed up for 6 months after delivery of the intervention.
Outcome data will be collected from pregnant women receiving antenatal care in the trial clusters, and women will be followed up for one year postnatally.

**Intervention type:** Other

**Primary outcome measures**

1. Recruitment rate of midwives attending the intervention training day in the intervention arm, calculated as percentage of eligible midwives consenting and attending the intervention (training) session
2. Feasibility of intervention delivery, calculated as the number of intervention sessions delivered with the planned number of midwives in attendance at each session (planned 6 midwives per session)
3. Intensity of intervention delivery, calculated as the number of intervention sessions required to deliver the intervention to all recruited midwives in the intervention arm at the end of intervention delivery
4. Time required for intervention delivery, calculated for all intervention sites as the number of weeks from the first contact with the site to arrange the delivery of the intervention, and the delivery of the final intervention session
5. Fidelity of intervention delivery, calculated as the frequency of the delivery of the intervention as planned measured by direct observation and video recording of the intervention sessions, and frequency of deviation from protocol

**Secondary outcome measures**

1. Process evaluation of the content, resources and delivery of the intervention, to be measured by:

1.1. Direct observations and video recordings of the intervention delivery for all intervention sessions
1.2. Participant evaluation forms to completed by midwives attending the intervention on the day of the intervention
1.3. Focus groups with a sample of midwives who have received the intervention, 1 month after intervention delivery

2. Process evaluation of the implementation of guidelines into routine midwifery practice following the intervention, to be measured by:

2.1. Midwife social cognitive theory questionnaires at baseline (pre-intervention) and 3 and 6 months after intervention delivery
2.2. Focus groups with a sample of midwives who have received the intervention, 1 month after intervention delivery

3. Process evaluation of women’s experience of midwifery care relating to their weight and implementation of midwifery advice and support into their own behaviours, among women with obesity (BMI≥30kg/m2) receiving care from midwives in the intervention arm, to be measured by:

3.1. A range of questionnaires in one pack for women to measure the advice received from their midwife about their weight and weight management, food frequency questionnaire, physical activity in pregnancy questionnaire, feelings about their weight, relationship with their midwife, and quality of life at baseline (3rd trimester)
3.2. Semi-structured interviews in the third trimester and at 6 months postnatal

4. Data required for sample size estimations for a future definitive trial will be measured by:

4.1. Calculating variance of the intervention primary and secondary outcome measures (data collection by questionnaires) for the midwives at baseline (pre-intervention) and follow up (3 and 6 months after intervention delivery)
4.2. Calculating variance of the intervention primary (weight measurement) and secondary (questionnaire) outcome measures for the pregnant women at baseline (3rd trimester) and follow up (3, 6, 9 and 12 months postnatal)
4.3. Rates of attrition of midwives and pregnant women who enrol in the study at the end of the study period

5. Data required to inform economic evaluation for a future definitive trial will be measured by an audit of routine antenatal data collection of participating women using handheld and electronic medical records to inform the variables to be included in the development of a framework for economic modelling, and variables not routinely recorded which require further data collection

**Overall trial start date:** 30/09/2015

**Overall trial end date:** 30/09/2017

**Participant inclusion criteria**

Midwives:
1. Midwives who have provided written informed consent for participation in the study prior to any study specific procedures
2. Community midwife role
3. Hospital-based midwife with a specific maternal obesity or weight management role

Pregnant women:
1. Pregnant women who have provided written informed consent for participation in the study prior to any study specific procedures
2. Booking body mass index ≥30kg/m2 (proxy measure for pre-pregnancy BMI)
3. Age at least 18 years (teenage pregnancies require specific nutritional support)
4. Singleton pregnancies (multiple pregnancies require specific nutritional support)
5. Women who have had their 12 week ultrasound scan and their pregnancy has progressed beyond the high risk period for miscarriage

**Participant type:** Patient

**Age group:** Adult

**Gender:** Both

**Target number of participants:** Planned Sample Size: 260; UK Sample Size: 260

**Participant exclusion criteria**

Midwives:
1. Hospital-based midwives without a specific maternal obesity or weight management role
2. Non-midwifery health professionals
3. Inability to speak/read English

Pregnant women
1. Pregnant women with a medical condition other than obesity which requires them to receive specialist weight management advice for that condition (e.g. women with pre-gestational diabetes who are attending a specialist antenatal diabetes clinic, pregnant women who have had bariatric surgery (e.g. gastric band or bypass) and require specialist nutritional support)
2. Pregnant women with substance misuse or known cause for concern (e.g. domestic violence)
3. Inability to speak/read English

**Recruitment start date:** 01/02/2016

**Recruitment end date:** 31/03/2017

**Locations**

**Countries of recruitment:** United Kingdom

**Trial participating centre:** Newcastle NHS Trust, The Royal Victoria Infirmary Queen, Victoria Road, Newcastle upon Tyne, NE1 4LP, United Kingdom

**Trial participating centre:** Gateshead NHT Trust, Queen Elizabeth Hospital Sherriff Hill
Gateshead, NE9 6SX, United Kingdom

**Trial participating centre:** Northumbria NHS Trust, North Tyneside General Hospital Rake Lane, North Shields, NE29 8NH, United Kingdom

**Trial participating centre:** South Tyneside NHS Trust, South Tyneside District Hospital, Harton Lane, South Shields, NE34 0PL, United Kingdom

**Sponsor information**

**Organisation:** The Newcastle Upon Tyne Hospitals NHS Foundation Trust

**Sponsor details:** Freeman Hospital, Freeman Road, High Heaton, Newcastle-Upon-Tyne, NE7 7DN, United Kingdom

**Sponsor type:** Hospital/treatment centre

**Funders**

**Funder type:** Government

**Funder name:** National Institute for Health Research

**Alternative name(s):** NIHR

**Funding Body Type:** government organisation

**Funding Body Subtype:** Federal/National Government

**Location:** United Kingdom

**Results and Publications**

**Publication and dissemination plan:** Planned publication of study protocol and results papers in a peer reviewed journal.

**Intention to publish date:** 31/12/2017

**Participant level data:** Available on request
