# Supplementary material for: GestationaL Obesity Weight management: Implementation of National Guidelines (GLOWING): a pilot cluster randomised controlled trial of a guideline implementation intervention for the management of maternal obesity by midwives
Source: Pilot Feasibility Stud. 2018 Feb 9;4:47. doi: 10.1186/s40814-018-0241-4 (PMC5807844; doi:10.1186/s40814-018-0241-4)
Supplement: Supplementary file 4 — GLOWING participant information sheets and consent forms for midwives (questionnaire data collection, participating in the intervention training days, focus groups) and pregnant women (baseline data collection, outcome data collection, interviews). (DOCX 218 kb) [file 40814_2018_241_MOESM4_ESM.docx]

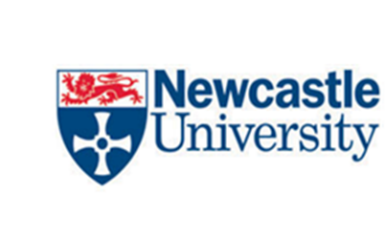


The GLOWING trial

Midwives’ Questionnaire

We would like to invite you to take part in a research study. Before you decide whether or not to take part it is important for you to understand what it will involve. Please take time to read the following information carefully and discuss it with others if you wish.

What is the purpose of the research?

Midwives have expressed the need for training and skills development to overcome some of the difficulties faced in their clinical practice relating to maternal obesity. The GLOWING study is a randomised controlled pilot study to see if a one day training intervention can support community midwives practice by addressing the training and development needs which midwives have identified. Midwives in two randomly allocated Trusts will be offered a one day training course as part of the research.

This information sheet relates to questionnaire-based data collection required for the four pilot study NHS Trusts. A random selection of midwives are being asked to complete questionnaires about experiences and current practice. The questionnaires will be completed at three time points (the first before any training is delivered, and a further two time points 3 and 6 months following training). Midwives will be requested to complete the questionnaires at each time point regardless of whether you receive training to explore if the responses from the midwives who received the training differ from those who did not. The questionnaires should take approximately 20 minutes to complete.

Why have I been invited?

You are a community midwife employed by a Trust that has agreed to participate in the study. You have been randomly selected to be invited to provide questionnaire data.

Do I have to take part?

It is up to you to decide whether you would like to participate in the study. We will describe the study in this information sheet and provide any further information you need. If you agree to take part we will then ask you to sign a consent form. You are free to withdraw at any time, without giving a reason.

What will happen to me if I take part?

If you agree to take part you will be asked to complete and return one questionnaire at the beginning of the study and two more, 3 and 6 months after the training has been delivered (regardless of whether you have received training).

What are the possible benefits and disadvantages and benefits of taking part?

You will need to give up some of your time to complete the questionnaires. There are no direct benefits for you but the information you provide will contribute valuable information to the results of this study and help to inform the design of future research on this topic.

Will my taking part in the study be kept confidential?

Yes. Your participation in the questionnaire-based data collection will not be disclosed to any of your clinical colleagues or management. The questionnaires will be stored securely, data will be stored on a password protected computer at Newcastle University, and all paper documents will be stored in a locked cupboard.

What would happen if I didn’t want to carry on with the study?

You can change your mind about taking part at any time. Any information already collected from you at the point of withdrawal will be retained for analysis (or destroyed if you wish).

What will happen to the results of the research study?

This is a pilot study and the results will be used to inform a larger national trial to assess whether this is an effective way to support midwives to implement weight management guidelines. The results may also be published in a journal. You would not be identified in any results we present or publish. If you would like to have further information about the results of the study sent to you then the research midwife can arrange for this to happen.

Who is organising and funding the research?

This project is being funded by the Department of Health via the National Institute for Health Research fellowship programme. It is being led by Dr Nicola Heslehurst, a Lecturer at Newcastle University.

Who has reviewed the study?

This study has been reviewed and given a favourable opinion by XXXXXX Research Ethics Committee.

**Where can I get further information about the study?**

If you have any questions or concerns about participating in the study please contact your local research midwife [insert local details], the study research midwife Cath McParlin (0191 2088239, [catherine.mcparlin@ncl.ac.uk](mailto:catherine.mcparlin@ncl.ac.uk)), or the lead research Dr Nicola Heslehurst (0191 2083823, [nicola.heslehurst@ncl.ac.uk](mailto:nicola.heslehurst@ncl.ac.uk)) who will be happy to answer any queries.

Thank you for taking the time to read this information.


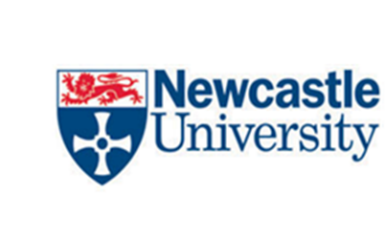


The GLOWING trial

Consent Form: Midwives Questionnaire

Study Identification Number**:**

Please read the statements below and **initial the boxes** to show that you agree with them.

| 1. | I confirm that I have read and understood the information sheet dated 30/09/2015 (Version 1) for the above study. I have had the opportunity to consider the information, ask questions, and have had these questions answered satisfactorily. |
| --- | --- |
| 2. | I understand that my participation is voluntary and that I am free to withdrawn at any time without giving any reason. |
| 3. | I understand that I will be asked to complete questionnaires at three separate time points. |
| 4. | I agree to take part in the above study |

_________________________________ __________ _______________________

Name of Participant Date Signature

_________________________________ __________ _______________________

Name of Health Professional/Researcher Date Signature

If you would like to receive a summary of the results of the questionnaire research by email or post, please provide the relevant address below:

Email:

__________________________________________________________________

Post:

__________________________________________________________________

__________________________________________________________________


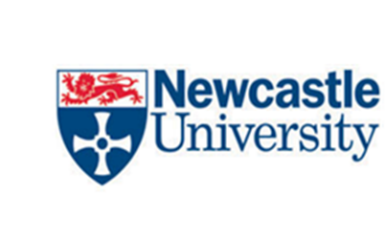


The GLOWING trial

Midwives training day

We would like to invite you to take part in a research study. Before you decide whether or not to take part it is important for you to understand what it will involve. Please take time to read the following information carefully and discuss it with others if you wish.

What is the purpose of the research?

NICE evidence-based guidelines for weight management during pregnancy include recommendations for health professionals to provide advice and support to obese women. Examples of recommendations include discussing obesity risks, diet and physical activity behaviours, incorporating practical and tailored advice, and being sensitive to women’s weight concerns.

Midwives have expressed the need for training and skills development to overcome some of the difficulties faced in their clinical practice. NICE have also identified professional development as a priority area in relation to maternal obesity, recommending that health professionals have the knowledge and skills to advise on weight management (including diet and physical activity behaviours), behaviour change, sensitive communication techniques, and knowledge of local services.

Therefore, we are carrying out this research to see if a one day training intervention can support community midwives practice by addressing the training and development needs which they have identified.

Why have I been invited?

We are inviting you to take part because you are a community midwife employed by [insert name] NHS Trust, which has agreed to participate in this research project.

Do I have to take part?

It is up to you to decide whether you would like to participate in the study. We will describe the study and provide any further information you need. If you agree to take part we will ask you to sign a consent form. You are free to withdraw at any time, without giving a reason.

What will happen to me if I take part?

If you agree to take part, the research team will contact you and invite you to attend a training session on a specified day. This will be arranged to suit you and your colleagues’ rota. The training will run between 8.30am and 4.30pm at a pre-identified venue. Refreshments, lunch and training resources will be provided, and any additional costs you incur by attending the day will be reimbursed. In order to assess the content, resources and delivery of the training the day will be video recorded and observed by the lead researcher on the study. You will also be asked to complete an evaluation form.

What are the possible benefits and disadvantages of taking part?

Hopefully you will find the training interesting and beneficial to your practice. It aims to develop your skills and knowledge, consolidating your previous experience and understanding. You can also use this training to contribute towards your CPD. However, you will need to engage in a full days training session instead of carrying out your normal clinical duties.

Will my taking part in the study be kept confidential?

No, your line manager and colleagues will be aware that you are attending the day as they will need to cover your workload. However, any recorded content, comments and your evaluation form will be treated confidentially. All the information collected during the research will be stored securely, video recordings will be transferred to a password protected computer at Newcastle University, and all paper documents will be stored in a locked cupboard.

What would happen if I didn’t want to carry on with the study?

You can change your mind about taking part at any time. Any information already collected from you at the point of withdrawal will be retained for analysis (or destroyed if you wish).

What will happen to the results of the research study?

This is a pilot study and the results will be used to inform a larger national trial to assess whether this is an effective way to support midwives to implement weight management guidelines. The results may also be published in a journal. You would not be identified in any results we present or publish. If you would like to have further information about the results of the study sent to you then the research midwife can arrange for this to happen.

Who is organising and funding the research?

This project is being funded by the Department of Health via the National Institute for Health Research fellowship programme. It is being led by Dr Nicola Heslehurst, a Lecturer at Newcastle University.

Who has reviewed the study?

This study has been reviewed and given a favourable opinion by XXXXXX Research Ethics Committee.

**Where can I get further information about the study?**

If you have any questions or concerns about participating in the study please contact your local research midwife [insert local details], the study research midwife Cath McParlin (0191 2088239, [catherine.mcparlin@ncl.ac.uk](mailto:catherine.mcparlin@ncl.ac.uk)), or the lead research Dr Nicola Heslehurst (0191 2083823, [nicola.heslehurst@ncl.ac.uk](mailto:nicola.heslehurst@ncl.ac.uk)) who will be happy to answer any queries.

Thank you for taking the time to read this information


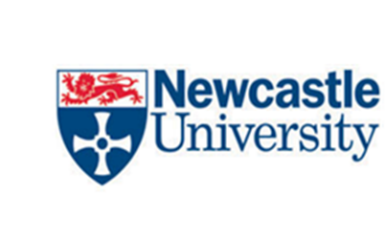


The GLOWING trial

Consent Form: Midwives intervention

Study Identification Number**:**

Please read the statements below and **initial the boxes** to show that you agree with them.

| 1. | I confirm that I have read and understood the information sheet dated 10/12/2015 (MWI Version 2) for the above study. I have had the opportunity to consider the information, ask questions, and have had these questions answered satisfactorily. |
| --- | --- |
| 2. | I understand that my participation is voluntary and that I am free to withdraw at any time without giving any reason. |
| 3. | I understand that the training will last one full day and I will be asked to complete an evaluation form. |
| 4. | I understand that the training I take part in will be video recorded and observed by a researcher. |
| 5.  6. | I understand that data collected during the study may be looked at by responsible individuals from regulatory authorities where it is relevant to this research study. I give permission for these individuals to have access to my data.  I agree to take part in the above study |

_________________________________ __________ ______________________

Name of Participant Date Signature

_________________________________ __________ _______________________

Name of Health Professional/Researcher Date Signature

If you would like to receive a summary of the training evaluation forms by email or post, please provide the relevant address below:

Email: __________________________________________________________________

Post: __________________________________________________________________


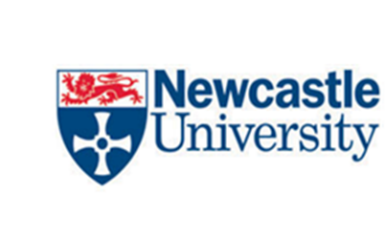


The GLOWING trial

Focus group evaluation

We would like to invite you to take part in a research study. Before you decide whether or not to take part it is important for you to understand what it will involve. Please take time to read the following information carefully and discuss it with others if you wish.

What is the purpose of the research?

This research aims to evaluate the GLOWING pilot study training day which you have attended, and identify if it can be refined for a future study. We want to explore your perspectives of the training content and delivery (e.g. if it was acceptable to you, whether anything was missing, views on the resources provided). We also want to explore how easy or difficult it has been to translate the training into your routine practice, and whether there is anything you have found particularly useful or that you would require further support with.

Why have I been invited?

You have been randomly selected from the group of midwives who have received the training.

Do I have to take part?

No, it is up to you to decide whether you would like to participate in this part of the study. We will describe the study in this information sheet and provide any further information you need. If you agree to take part we will then ask you to sign another consent form. You are free to withdraw at any time, without giving a reason.

What will happen to me if I take part?

If you agree to take part you will be invited to attend a focus group with around 3-4 other midwives from your NHS Trust who attended one of the training days. The group will be organised and led by Dr Nicola Heslehurst who is the lead researcher on the study, and may be observed by another member of the research team. During the focus group you would be asked to discuss the training day, what was good or not so good, what was useful and what was missing and could be added. You can give as much or as little information as you would like during the focus group. The focus group discussion will be audio recorded.

What are the possible disadvantages and benefits of taking part?

You will need to give up some of your time if you agree to take part but this should happen during working hours. There are no direct benefits for you but the information you provide will help to refine the training day and inform future research.

Will my taking part in the study be kept confidential?

Your manager and colleagues may be aware that you are taking part in the focus groups if they need to cover your workload, or if they are also participating in the focus group. However, any recorded content and comments will be treated confidentially by the research team. All the information collected during the research will be stored securely, recordings will be transferred to a password protected computer at Newcastle University, and all paper documents will be stored in a locked cupboard.

What would happen if I didn’t want to carry on with the study?

You can change your mind about taking part at any time up to participation in the focus group, and you can withdraw from participating any further during the focus group. However, the nature of focus groups means that information you have provided would be impossible to withdraw as it might influence the direction of the discussion and other participants’ comments. Therefore any information already collected from you at the point of withdrawal will be included in the analysis.

What will happen to the results of the research study?

This is a pilot study and the results will be used to inform a larger national trial to assess whether this is an effective way to support midwives to implement weight management guidelines. The results may also be published in a journal. You would not be identified in any results we present or publish. If you would like to have further information about the results of the study sent to you then the research midwife can arrange for this to happen.

Who is organising and funding the research?

This project is being funded by the Department of Health via the National Institute for Health Research fellowship programme. It is being led by Dr Nicola Heslehurst, a Lecturer at Newcastle University.

Who has reviewed the study?

This study has been reviewed and given a favourable opinion by XXXXXX Research Ethics Committee.

**Where can I get further information about the study?**

If you have any questions or concerns about participating in the study please contact your local research midwife [insert local details], the study research midwife Cath McParlin (0191 2088239, [catherine.mcparlin@ncl.ac.uk](mailto:catherine.mcparlin@ncl.ac.uk)), or the lead research Dr Nicola Heslehurst (0191 2083823, [nicola.heslehurst@ncl.ac.uk](mailto:nicola.heslehurst@ncl.ac.uk)) who will be happy to answer any queries.

Thank you for taking the time to read this information.


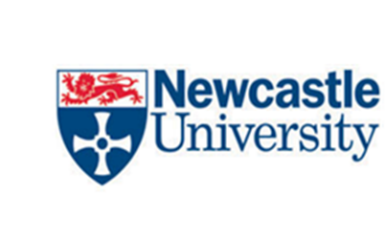


The GLOWING trial

Consent Form: Midwives Focus Group

Study Identification Number**:**

Please read the statements below and **initial the boxes** to show that you agree with them.

| 1. | I confirm that I have read and understood the information sheet dated 10/12/2015 (MWFG Version 2) for the above study. I have had the opportunity to consider the information, ask questions, and have had these questions answered satisfactorily. |
| --- | --- |
| 2. | I understand that my participation is voluntary and that I am free to withdraw at any time without giving any reason. |
| 3. | I understand that the focus group will be audio recorded and the researcher may make a record of any comments made. |
| 4. | I understand that data collected during the study may be looked at by responsible individuals from regulatory authorities where it is relevant to this research study. I give permission for these individuals to have access to my data. |
| 5. | I agree to take part in the above study |

_____________________________ __________ _______________________

Name of Participant Date Signature

_________________________________ __________ _______________________

Name of Health Professional/Researcher Date Signature

If you would like to receive a summary of the focus group results by email or post, please provide the relevant address below:

Email: __________________________________________________________________

Post: __________________________________________________________________

__________________________________________________________________


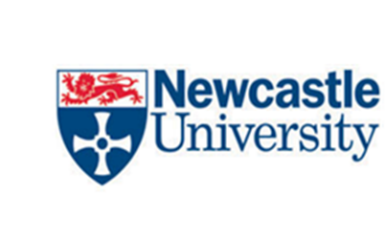


The GLOWING trial

Women’s Questionnaire and Weight Measurement

We would like to invite you to take part in a research study. Before you decide whether or not to take part it is important for you to understand what it will involve. Please take time to read the following information carefully and discuss it with others if you wish.

What is the purpose of the research?

This research is part of the GLOWING trial. The GLOWING trial is providing a specific training package to community midwives, and then assessing whether the training changes the type of advice and support midwives give to pregnant women in their routine clinical practice. The training package aims to support community midwives to provide the best care and support for women who have a body mass index (BMI) over 30.

The study is being carried out in 4 hospitals in the North East of England, but only community midwives from 2 of the hospitals will be provided with the training. We will be comparing the type of advice and support provided by midwives who have received the training with those who have not had any training to see if there is a difference. Before we start the training sessions, we would like to make sure that there are no differences in the characteristics of women who book for their care at the four different hospitals.

Why have I been invited?

You have been invited to take part in the study because you have had a booking appointment with a community midwife from one of the 4 hospitals involved in the GLOWING trial. Out of all of the women who had a booking BMI over 30 in the participating hospitals, we have randomly selected 72 women to invite to take part in this study.

Do I have to take part?

No, it is up to you to decide whether you would like to participate in the study. We will describe the study in this information sheet and provide any further information you need. If you agree to take part we will then ask you to sign a consent form. You are free to withdraw at any time, without giving a reason. If you do not wish to take part your care will not be affected in any way.

What will happen to me if I take part?

If you agree to take part you will be asked to complete and return a questionnaire and to be weighed by the research midwife once between 28 and 40 weeks of pregnancy.

What are the possible disadvantages and benefits of taking part?

You will need to give up some of your time to complete the questionnaire. There are no direct benefits for you but the answers you provide will contribute valuable information to the results of this study and help to plan future research.

Will my taking part in the study be kept confidential?

Yes. The questionnaires and all the information collected will be stored securely, data will be stored on a password protected computer at Newcastle University, and all paper documents will be stored in a locked cupboard. All information collected from you will be kept confidential and none of the information you provide to the researchers will be seen by your care team (e.g. midwife or obstetrician).

What would happen if I didn’t want to carry on with the study?

You can change your mind about taking part at any time. Any information already collected from you can be destroyed if you wish. This will not affect the care you receive in any way.

**Expenses and Payments**

We do not anticipate that this study will create any expense to you but if it does you will be reimbursed. We will give you a £10 shopping voucher to thank you for taking part.

What will happen to the results of the research study?

The results from the questionnaires will be used to see if there are any differences in the characteristics of women having care at the four hospitals taking part in the GLOWING trial. The results from the GLOWING trial may be published in a scientific journal so that other researchers and health professionals can learn from the information we collect. You would not be identified in any results we present or publish. If you would like to receive a summary of results from the study then the research midwife can arrange for this to happen.

Who is organising and funding the research?

This project is being funded by the Department of Health via the National Institute for Health Research. It is being led by Nicola Heslehurst who is a researcher at Newcastle University.

Who has reviewed the study?

This study has been reviewed and given a favourable opinion by XXXXXX Research Ethics Committee.

**Where can I get further information about the study?**

If you have any questions or concerns about participating in the study please contact your local research midwife [insert local details], the study research midwife Cath McParlin (0191 2088239, [catherine.mcparlin@ncl.ac.uk](mailto:catherine.mcparlin@ncl.ac.uk)), or the lead research Nicola Heslehurst (0191 2083823, [nicola.heslehurst@ncl.ac.uk](mailto:nicola.heslehurst@ncl.ac.uk)) who will be happy to answer any queries.

Thank you for taking the time to read this information.


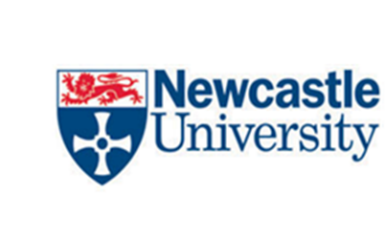


The GLOWING trial

Consent Form: Women’s Questionnaire and Weight Measurement (Baseline)

Study Identification Number**:**

Please read the statements below and **initial the boxes** to show that you agree with them.

| 1. | I confirm that I have read and understood the information sheet dated 10/12/2015 (WQB Version 2) for the above study. I have had the opportunity to consider the information, ask questions, and have had these questions answered satisfactorily. |
| --- | --- |
| 2. | I understand that my participation is voluntary and that I am free to withdraw at any time without giving any reason, without my medical care or legal rights being affected. |
| 3. | I understand that relevant sections of my medical notes and data collected during the study, may be looked at by individuals from the [insert local NHS Trust] or from regulatory authorities where it is relevant to my taking part in this research. I give permission for these individuals to have access to my records. |
| 4. | I agree to take part in the above study |

________________________________ __________ _______________________

Name of Participant Date Signature

_________________________________ __________ _______________________

Name of Health Professional/Researcher Date Signature

If you would like to receive a summary of the results by email or post, please provide the relevant address below:

Email: __________________________________________________________________

Post: __________________________________________________________________

__________________________________________________________________


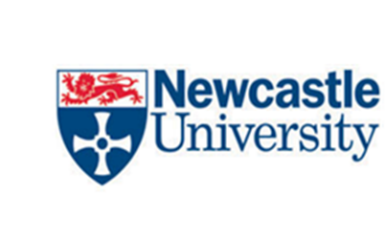


The GLOWING Trial Participant Information Sheet

Women’s Questionnaires and Weight Measurements

We would like to invite you to take part in a research study. Before you decide whether or not to take part it is important for you to understand what it will involve. Please take time to read the following information carefully and discuss it with others if you wish.

What is the purpose of the research?

This research is part of the GLOWING trial. The GLOWING trial is providing a specific training package to community midwives, and then assessing whether the training changes the type of advice and support midwives give to pregnant women in their routine clinical practice. The training package aims to support community midwives to provide the best care and support for women who have a body mass index (BMI) over 30.

The study is being carried out in 4 hospitals in the North East of England, but only community midwives from 2 of the hospitals will be provided with the training. We will be comparing the type of advice and support provided by midwives who have received the training with those who have not had any training to see if there is a difference. We also want to compare whether there is any difference between the pregnant women who the midwives care for.

Why have I been invited?

You have been invited to take part in the study because you have had a booking appointment with a community midwife from one of the 4 hospitals involved in the GLOWING trial. Out of all of the women who had a booking BMI over 30 in the participating hospitals, we have randomly selected 72 women to invite to take part in this study.

Do I have to take part?

No, it is up to you to decide whether you would like to participate in the study. We will describe the study in this information sheet and provide any further information you need. If you agree to take part we will then ask you to sign a consent form. You are free to withdraw at any time, without giving a reason. If you do not wish to take part your care will not be affected in any way.

What will happen to me if I take part?

If you agree to take part you will be asked to complete and return a questionnaire and to be weighed by the research midwife once between 28 and 40 weeks of pregnancy. The research midwife will then arrange to see you when your baby is 3, 6, 9 and 12 months old. At this visit she will ask you to complete another questionnaire, weigh you and collect information, such as weight, from your babies red ‘child health record’ book. We would also like to look at your hospital records to collect information about your health and wellbeing during pregnancy and the type of delivery you had.

What are the possible disadvantages and benefits of taking part?

You will need to give up some of your time to complete the questionnaires and to be weighed. There are no direct benefits for you but the answers you provide will contribute valuable information to the results of this study and help to plan future research.

Will my taking part in the study be kept confidential?

Yes. The questionnaires and all the information collected will be stored securely, data will be stored on a password protected computer at Newcastle University, and all paper documents will be stored in a locked cupboard. All information collected from you will be kept confidential and none of the information you provide to the researchers will be seen by your care team (e.g. midwife or obstetrician).

What would happen if I didn’t want to carry on with the study?

You can change your mind about taking part at any time. Any information already collected from you can be destroyed if you wish. This will not affect the care you receive in any way.

**Expenses and Payments**

We do not anticipate that this study will create any expense to you, but if it does you will be reimbursed. We will give you a £10 shopping voucher every time you complete a questionnaire to thank you for your time (maximum of £50 shopping vouchers).

What will happen to the results of the research study?

The results from the questionnaires and weight measurements will be used to see if providing training to the midwives has any effect on your health and wellbeing. The results from the GLOWING trial may be published in a scientific journal so that other researchers and health professionals can learn from the information we collect. You would not be identified in any results we present or publish. If you would like to receive a summary of results from the study then the research midwife can arrange for this to happen.

Who is organising and funding the research?

This project is being funded by the Department of Health via the National Institute for Health Research. It is being led by Nicola Heslehurst who is a researcher at Newcastle University.

Who has reviewed the study?

This study has been reviewed and given a favourable opinion by XXXXXX Research Ethics Committee.

**Where can I get further information about the study?**

If you have any questions or concerns about participating in the study please contact your local research midwife [insert local details], the study research midwife Cath McParlin (0191 2088239, [catherine.mcparlin@ncl.ac.uk](mailto:catherine.mcparlin@ncl.ac.uk)), or the lead research Nicola Heslehurst (0191 2083823, [nicola.heslehurst@ncl.ac.uk](mailto:nicola.heslehurst@ncl.ac.uk)) who will be happy to answer any queries.

Thank you for taking the time to read this information.


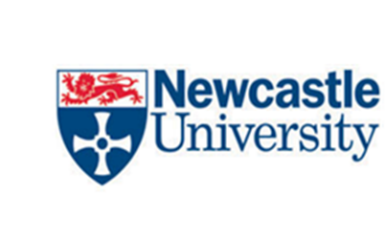


The GLOWING Trial

Consent Form: Women’s Questionnaire and Weight Measurement

Study Identification Number**:**

Please read the statements below and **initial the boxes** to show that you agree with them.

| 1. | I confirm that I have read and understood the information sheet dated 10/12/2015 (WQ Version 2) for the above study. I have had the opportunity to consider the information, ask questions, and have had these questions answered satisfactorily. |
| --- | --- |
| 2. | I understand that my participation is voluntary and that I am free to withdraw at any time without giving any reason, without my medical care or legal rights being affected. |
| 3. | I understand that relevant sections of my medical notes and data collected during the study, may be looked at by individuals from the [insert local NHS Trust] or from regulatory authorities where it is relevant to my taking part in this research. I give permission for these individuals to have access to my records. |
| 4. | I agree to being contacted after the birth of my baby for study follow up appointments (3, 6, 9 and 12 months). |
| 5. | I agree to take part in the above study |

____________________________ __________ _______________________

Name of Participant Date Signature

_______________________________ __________ _______________________

Name of Health Professional/Researcher Date Signature

Email: __________________________________________________________________

Post: __________________________________________________________________

__________________________________________________________________


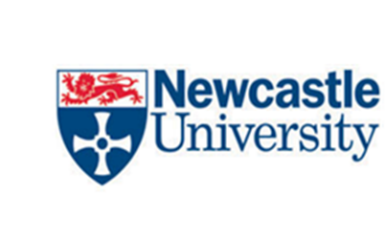


The GLOWING Trial Participant Information Sheet

Women’s Interviews

We would like to invite you to take part in a research study. Before you decide whether or not to take part it is important for you to understand what it will involve. Please take time to read the following information carefully and discuss it with others if you wish.

What is the purpose of the research?

This research is part of the GLOWING trial. The GLOWING trial is providing a specific training package to community midwives, and then assessing whether the training changes the type of advice and support midwives give to pregnant women in their routine clinical practice. The training package aims to support community midwives to provide the best care and support for women who have a body mass index (BMI) over 30.

Why have I been invited?

You have been invited to take part in the study because you have already consented to provide questionnaire and weight measurements for GLOWING. Out of all of the women already participating in GLOWING, we have randomly selected 28 women to invite to participate in an extra part of the study.

Do I have to take part?

No, it is up to you to decide whether you would like to participate in the study. We will describe the study in this information sheet and provide any further information you need. If you agree to take part we will then ask you to sign a consent form. You are free to withdraw at any time, without giving a reason. If you do not wish to take part your care will not be affected in any way.

What will happen to me if I take part?

If you agree to take part you will be contacted by someone from the research team to arrange a date and time to visit you to ask you some questions (an interview). This will happen after 28 weeks into your pregnancy, and the visit can happen at a place of your choosing (e.g. work, home). The researcher who visits you will ask you a series of questions on your opinions about the care, information and advice you have received about healthy lifestyle habits from your community midwife/midwives.

You will also be contacted when your baby is 6 months old and invited to participate in a second interview to get your opinions about healthy lifestyles after having your baby. To ensure all information is collected accurately the interview would be recorded using a small audio-recorder. The length of the interview varies but most interviews last between 30 and 60 minutes.

What are the possible disadvantages and benefits of taking part?

You will need to give up some of your time for the interview. There are no direct benefits for you but the answers you provide will contribute valuable information to the results of this study and help to plan future research.

Will my taking part in the study be kept confidential?

Yes. The interview would be recorded onto a digital audio recorder and transferred to a password protected computer at Newcastle University. This would only be accessible to the researcher who did the interview and the research secretary who will type up a transcript of the interview (a word for word account of what was said) before deleting the audio file from the computer. The researcher would remove any names that were used during the interview from the transcript.

Rules about research mean that we would need to keep a copy of the transcript for 10 years after the research is finished. The transcript would be kept in a secure location and would only be accessible by the research team.

What would happen if I didn’t want to carry on with the study?

You can change your mind about taking part at any time. Any information already collected from you can be destroyed if you wish. This will not affect the care you receive in any way.

**Expenses and Payments**

Any expenses incurred to attend the interviews will be reimbursed. We will give you a £10 shopping voucher following each interview as a thank you for your time (maximum of £20 shopping vouchers). These vouchers are in addition to the shopping vouchers you will receive for returning the questionnaires.

What will happen to the results of the research study?

The results from the questionnaires and weight measurements will be used to see if providing training to the midwives has any effect on your health and wellbeing. The results from the GLOWING trial may be published in a scientific journal so that other researchers and health professionals can learn from the information we collect. You would not be identified in any results we present or publish. If you would like to receive a summary of results from the study then the research midwife can arrange for this to happen.

Who is organising and funding the research?

This project is being funded by the Department of Health via the National Institute for Health Research. It is being led by Nicola Heslehurst who is a researcher at Newcastle University.

Who has reviewed the study?

This study has been reviewed and given a favourable opinion by XXXXXX Research Ethics Committee.

**Where can I get further information about the study?**

If you have any questions or concerns about participating in the study please contact your local research midwife [insert local details], the study research midwife Cath McParlin (0191 2088239, [catherine.mcparlin@ncl.ac.uk](mailto:catherine.mcparlin@ncl.ac.uk)), or the lead research Dr Nicola Heslehurst (0191 2083823, [nicola.heslehurst@ncl.ac.uk](mailto:nicola.heslehurst@ncl.ac.uk)) who will be happy to answer any queries.

Thank you for taking the time to read this information.


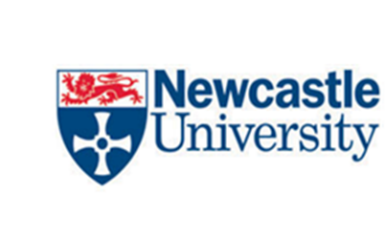


The GLOWING Trial

Consent Form: Women’s Interview

Study Identification Number**:**

Please read the statements below and **initial the boxes** to show that you agree with them.

| 1. | I confirm that I have read and understood the information sheet dated 10/12/2015 (WI Version 2) for the above study. I have had the opportunity to consider the information, ask questions, and have had these questions answered satisfactorily. |
| --- | --- |
| 2. | I understand that I will be contacted after 28 weeks of pregnancy for the first interview, and when my baby is 6 months old for a second interview. |
| 3. | I understand that my participation is voluntary and that I am free to withdraw at any time without giving any reason, without my medical care or legal rights being affected. |
| 4. | I understand that relevant sections of my medical notes and data collected during the study, may be looked at by individuals from the [insert local NHS Trust] or from regulatory authorities where it is relevant to my taking part in this research. I give permission for these individuals to have access to my records. |
| 5. | I understand that the interview will be audio recorded |
| 6. | I agree to take part in the above study |

_________________________________ __________ _______________________

Name of Participant Date Signature

_________________________________ __________ _______________________

Name of Health Professional/Researcher Date Signature

If you would like to receive a summary of the results by email or post, please provide the relevant address below:

Email: __________________________________________________________________

Post: _________________________________________________________________
